# Evaluation of Conidia-Dust Formulation of the Entomopathogenic Fungus, *Metarhizium anisopliae* to Biocontrol the Brown-Banded Cockroach, *Supella longipalpa* F.

**DOI:** 10.5812/jjm.10721

**Published:** 2014-06-01

**Authors:** Mona Sharififard, Mohammad Saeed Mossadegh, Babak Vazirianzadeh, Seyed Mahmood Latifi

**Affiliations:** 1 Department of Medical Entomology and Vector Control, School of Health, Ahvaz Jundishapur University of Medical Sciences, Ahvaz, IR Iran; 2 Department of Plant Protection, College of Agiculture, Chamran Univercity, Ahvaz, IR Iran; 3 Department of Medical Entomology and Vector Control, School of Health and Infectious and Tropical Disease Research Centre, Ahvaz Jundishapur University Medical Sciences, Ahvaz, IR Iran; 4 Department of Statics and Epidemiology, School of Health, Ahvaz Jundishapur University of Medical Sciences, Ahvaz, IR Iran

**Keywords:** Supella Longipalpa, Biological Control, *Metarhizium anisopliae*

## Abstract

**Background:**

The brown-banded cockroach *Supella longipalpa* (F.) as a mechanical vector of pathogens and source of allergens has recently become widespread in the city of Ahvaz, southwestern Iran.

**Objectives:**

This research was done to evaluate the efficacy of a dust-formulation of *Metarhizium anisopliae* isolate IRAN 437C, as a common entomopathogenous fungus, against *S. longipalpa*.

**Materials and Methods:**

Conidia dust-formulations of *M. anisopliae* were prepared in proportions of 1%, 5%, 10%, 25%, 50% and 100% with bad wheat flour as the carrier. Cockroaches were exposed to surfaces treated with 1.5 mg/cm^2^ of the formulations under laboratory and semi-field conditions.

**Results:**

Cockroach mortality rates increased and survival times (ST_50_) decreased with an increased proportion of conidia from 1% to 100% but records taken for mortality and survival time from proportions of 25%, 50% and 100% were not significantly different. The mortality rates reached 100% and 90-100% in adults and nymphs, respectively on the seventh day. The lowest ST_50_ was related to the proportion of 100% (3 days). Probit analysis indicated LD_50_ and LD_90_ values of 1.7 × 10^6^ and 1.7 × 10^7^ conidia/cm^2^ for adults and these values changed to 4.5 × 10^6^ and 2.9 × 10^7^ for third and fourth instar nymphs at three days post exposure. Proportion of 25% caused mortality rates of 87%, 81% and 73% in adult, adult & nymph and nymph populations, respectively at four days after exposure under room conditions.

**Conclusions:**

Conidia dust-formulation of *M. anospliae* isolate IRAN 437C could present a promising alternative to control the brown-banded cockroach.

## 1. Background

Cockroaches have long been known as vectors of food poisoning and infectious organisms (1-3). The brown-banded cockroach, *Supella longipalpa* (F.) can be considered as a nearly cosmopolitan cockroach (1-4). It is now distributed throughout tropical and subtropical regions of the world (1). This cockroach species carries a variety of microorganisms, as a vector for pathogenic bacteria in urban environments and it has also been reported as a source of allergens (3, 5, 6). It is commonly found in homes, apartments, hotels and hospitals and less frequently in stores, restaurants and kitchens (1, 2, 7). The brown-banded cockroaches do not require as much moisture as the German cockroach, *Blattela germanica*, so they are normally found in rooms other than those containing wet areas such as kitchens or bathrooms. This widespread habitat of the brown-banded cockroach distributed throughout different areas of homes and buildings makes them very difficult to control (7).

Cockroaches are traditionally controlled by applications of liquid formulations of pyrethroids, carbamates or organophosphates in or nearby infested harborages (7). This method is difficult to apply in case of *S. longipalpa* infestations because it is necessary to treat all the different areas of a room that the cockroaches may inhabit without creating pollution problems, which cause damage to furniture and other points where this insect prefers to glue its ootheca (1, 7, 8). The various risk factors associated with the use of chemical insecticides such as the development of resistance, associated resurgence in insects, accumulation of pesticide residue in a food chain, environmental pollution, health risks and high costs have driven scientists and farmers to develop alternative strategies for pest control (8, 9). The entomopathogenic fungus, *Metarhizium anisopliae* (Metch.) Sorokin has been isolated from 200 insect species including the orders Lepidoptera, Coleoptera, Orthoptera and Hemiptera (10). This fungus was produced commercially as a bio-path by Ecoscience to control the German cockroach, *B. germanica* (11).

There are very rare examples of using other species of fungi against *S. longipalpa*. One of them is using *Beauveria bassiana* (the chines isolte B.C.) and *Trichoderma harzianum* to control *S. longipalpa* in Iraq (12). Numerous studies have been done to control the German cockroach as the most important cockroach species responsible for transferring pathogens and for the development of resistance to chemical insecticides, by entomopathogenic fungi (11, 13-17); however, there is no documented study related to the control of the brown-banded cockroach by entomopathogenic fungi, thus the current research was done to evaluate the efficacy of conidia dust- formulation of *M. anisopliae* isolate IRAN 437C against the brown-banded cockroach. This cockroach species has recently been widely distributed in residential homes especially in apartments and has become a serious health problem in Ahvaz, Khozestan province, southwestern Iran.

## 2. Objectives

This research was done to evaluate the efficacy of a dust-formulation of *M. anisopliae* isolate IRAN 437C, as a common entomopathogenous fungus, against *S. longipalpa*.

## 3. Materials and Methods

Insects: The cockroaches were reared in plexiglas containers (25 cm high × 17 cm diameter) and maintained at 27 ± 2˚C, 50 ± 5% RH for a photoperiod of 12:12 (L:D) for nearly one year. They were fed with dry crumble biscuits, bread and water. Pieces of facial tissue were provided as harborage. Cockroaches were anesthetized by chilling to facilitate handling.

### 3.1. Fungus

Four strains of entomopathogenic fungus, *M. anisopliae* strain Iran 437C, *M. anisopliae* strain Iran 715C, *M. anisopliae* strain 1018, *M. anisopliae* strain Rhynchophorus, were provided by the fungi collection of the Plant Protection Institute of Iran. They were cultured on SDAY plates, kept at 27˚C, RH: 75 ± 5 % and in photoperiod condition of 12:12 (L: D) hours. Sporulating cultures were harvested by scraping dry conidia from the surface of the culture plate with a scalpel.

### 3.2. Conidia Drying

After harvesting, conidia were flattened on a filter paper and the filter paper was replaced daily for a period of three days. Conidia were then sieved with a tea strainer to remove the medium particles. The resulting conidia powder was then mixed evenly with the carrier, bad wheat flour. Numbers of conidia per gram of net conidia-powder were determined using a hemocytometer before being mixed with flour. This was determined as 4.4 × 10^7^ conidia per gram powder.

#### 3.2.1. Bioassay 1: Treated Surface: Small scale

The least amount of conidia dust-formulation required to cover an entire surface was recorded in a pretest. Then formulations were made in proportions of 1%, 5%, 10%, 25%, 50% and 100% of conidia with bad wheat flour as the carrier (proportions were equal to 6.6 × 10^5^, 3.3 × 10^6^, 6.6 × 10^6^, 1.65 × 10^6^, 3.3 × 10^7^ and 6.6 × 10^7^ conidia/cm^2^). Bottoms of glass jars (≈ 64 cm^2^) were treated with 1.5 mg/cm^2^ of dust-formulation spread homogenously with a brush. The inner surfaces of jars were smeared with a thin layer of butter to restrict cockroach movement on the bottom. Groups of 20 adults (10 females and 10 males) or 20 older nymphs were anesthetized by chilling and transferred to plastic cups. After recovery, they were transferred to treated glass jars. Insects were kept confined for one hour in contact with treated surfaces. Then they were transferred to safe containers and fed with bread and water. The control group was exposed to the surface treated with 1.5 mg/cm^2^ of bad wheat flour. Treated cockroaches were kept under laboratory conditions (25 ± 1 **°**C, RH: 75 ± 5% and a photoperiod of 12:12). Cockroach mortality was checked on the third and seventh day. Cadavers were collected, surface sterilized and transferred to sterile plates containing a damp filter paper. Occurrence of true mortality was taken for those cadavers in which sporulation were visible. There were four replicates for each treatment.

#### 3.2.2. Bioassay 2: Treated Surface: Larger Scale (Semi-field Conditions)

The best proportion of conidia-dust formulation in terms of causing the highest cockroach mortality in the shortest time and using less conidium was selected from the previous experiment (proportions of 10% and 25%). Five fluorescent coatings (1 m^2^) were prepared and the inner surfaces of walls were smeared with a thin layer of butter. The bottom of these coatings was treated with 1.5 mg/cm^2^ of conidia-dust, homogenously spread with a brush. Cockroaches (100 older nymphs, adults or combination of nymphs and adults) were released simultaneously from rearing containers into the treated surfaces. The control group was exposed to surfaces treated with 1.5 mg/cm^2^ of bad wheat flour. Water, food and pieces of cardboard were provided for rest. Treated cockroaches were kept under room conditions. This experiment was repeated four times. Mortality was checked on the 3rd and seventh day.

### 3.3. Data Analysis

Mortality percentages were corrected using Abbott’s (1925) formula and submitted to probit analysis to determine lethal doses (LD_50_ and LD_90_). Survival analysis was applied in order to estimate mean survival times (ST_50_) and their 95% fiducially limits (FL95%) were determined by the Kaplan-Mayer method. Survival and probit analyses were determined by the SPSS software (version 16). Analyses by ANOVA and comparison of mortality percentage means was done in a completely randomized design by Tukey’s test (P < 0.05), using the SAS software (version 9.1.3).

## 4. Results

### 4.1. Bioassay 1: Treated Surface: Small Scale

Results of pretests done for screening four *M. anisopliae* isolates indicated that *M. anisopliae* Iran 437C had a high virulence against *S. longipalpa*. Adult mortality increased with increased conidia proportions in dust-formulations from 1% to 100% but there was no significant difference between proportions of 25%, 50% and 100% in terms of percentage mortality (Table 1). Adult mortality was treated with the lowest and highest proportions of conidia ranging from 35% to 100% at three days and 52.5-100% at seven days after exposure. Mortality was 100% for all the three proportions of 25% (1.65 × 10^7^ conidia/cm^2^), 50% (3.3 × 10^7^ conidia/cm^2^) and 100% (6.6 × 10^7^ conidia/cm^2^) on the seventh day. For the control group, percentage mortality was lower than 5% for the duration of the experiment (one week) and it was significantly different from all the treated groups (Table 1).

Probit analysis indicated values of 1.7 × 10^6^ and 1.7 × 10^7^ conidia/cm^2^ for LD_50_ and LD_90_ at three days after exposure and these values were 7.8 × 10^5^ and 8.1 × 10^6^ conidia/cm^2^ at seven days after exposure for adults (Table 2). Also, survival time analysis (ST_50_) showed a significant difference between ST_50_ of adults from treated surfaces compared to untreated surfaces or those in the control group. Shortest adult survival time belonged to the proportion of 100% after three days but ST_50_ values at proportions of 25% and 50% were not significantly different because their fiducial limits completely overlapped (Table 3).

**Table 1.** Cumulative Mortality (%) of *S. longipalpa* Nymphs and Adults Exposed to Different Doses of *M. anisoplae* (IRAN 437C) Presented as Powder Formulation at Small Scale (27 ± 1, RH > 60%, 12:12 photoperiod.) ^a^

*M. anisopliae*-Powder Formulation, conidia/cm^2^	Nymphs			Adults
	Days After Inoculation			
	3 Days	7 Days	3 Days	7 Days
**Control (bad wheat flour)**	0 ± 0.0	2. 5 ± 1.4	0 ± 0.0	1.2 ± 1.2
**1% (6.6×10** ^**5)**^	32 ± 4.3	45 ± 4.1	35 ± 3.5	52.5 ± 4.3
**5% (3.3×10**^**6**^)	57.5 ± 4.3	69 ± 2.4	60 ± 3.5	72.5 ± 4.3
**10% (6.6×10** ^**6)**^	72.5 ± 4.8	82.5 ± 4.3	77.5 ± 4.8	85 ± 4.1
**25% (1.65×10** ^**7)**^	85 ± 2.04	92.5 ± 3.2	90 ± 2.1	100 ± 0.0
**50% (3.3×10** ^**7)**^	90 ± 2.04	97.5 ± 2.5	93.7 ± 4.8	100 ± 0.0
**100% (6.6×10** ^**7)**^	100 ± 0.0	100 ± 0.0	100 ± 0.0	100 ± 0.0
**ANOVA**	P < 0.0001	P < 0.0001	P < 0.0001	P < 0.0001

^a^ Mean ± SD (Tukey’s test α = 0.05).

**Table 2.** Probit Analysis of *M. anisopliae* (IRAN 437C) Conidia-dust Formulation Against Adults and Nymphs of *S. longipalpa* Presented as Treated Surface

-	LD_50_ (95% CI^a^)	LD_90_ (95% CI)	No. (df)
**Adults, 3d**	1.7 × 10^6^ (1.1×10^6^-2.2 × 10^6^)	1.7 × 10^7^ (1.2 × 10^7^-2.6 × 10^7^)	13.3 (22)
**Nymphs, 3d**	4.5 × 10^6^ (1.3 × 10^6^-8.2. ×10^6^)	2.9 × 10^7^ (1.9 × 10^7^-4.6 × 10^7^)	13 (21)
**Adults, 7d**	7.8 × 10^5^ (6.5 × 10^5^-1.1 × 10^6^)	3.1 × 10^6^ (3.8 × 10^5^-6.5 × 10^6^)	15.9 (14)
**Nymphs, 7d**	3.1 × 10^6^ (3.8 × 10^5^-6.5 × 10^6^)	8.1 × 106 (5.5 × 10^6^-1.4 × 10^7^)	15.1 (17)

^a^ Abbreviation: CI, confidence interval.

**Table 3.** Mean Survival Times of *S. longipalpa* Nymphs and Adults Exposed to *M. anisopliae *(IRAN 437C) Presented as Powder Formulation After Seven Days (27 ± 1, RH > 60%, 12:12 photoperiod)

*M. anisopliae*- Powder Formulation, conidia/cm^2^	Nymphs		Adults	
	**Mean ± SE**	**FL (95%)**	**Mean ± SE**	**FL** ^ a^ ** (95%)**
**Control (bad wheat flour)**	6.7 ± 0.1	6.8-7.1	6.8 ± 0.3	6.2-7.4
**1%, 6.6 × 10** ^**5**^	5.7 ± 0.21	5.3-6.2	5.6 ± 0.21	5.2-6.02
**5%, 3.3 × 10** ^**6**^	4.7 ± 0.22	4.3-5.1	4.6 ± 0.22	4.2-5.03
**10%, 6.6 × 10** ^**6**^	4.1 ± 0.2	3.7-4.5	3.9 ± 1.9	3.5-4.3
**25%, 1.65 × 10** ^**7**^	3.6 ± 0.16	3.3-3.9	3.4 ± 0.13	3.1-3.7
**50%, 3.3 × 10** ^**7**^	3.4 ± 0.13	3.1-3.7	3.2 ± 0.11	3.04-3.7
**100% ( 6.6 × 10** ^**7**^ **)**	3 ± 0.00	-	3 ± 0.00	-

^a^ Abbreviation: FL, fiducially limits.

**Figure 1.**
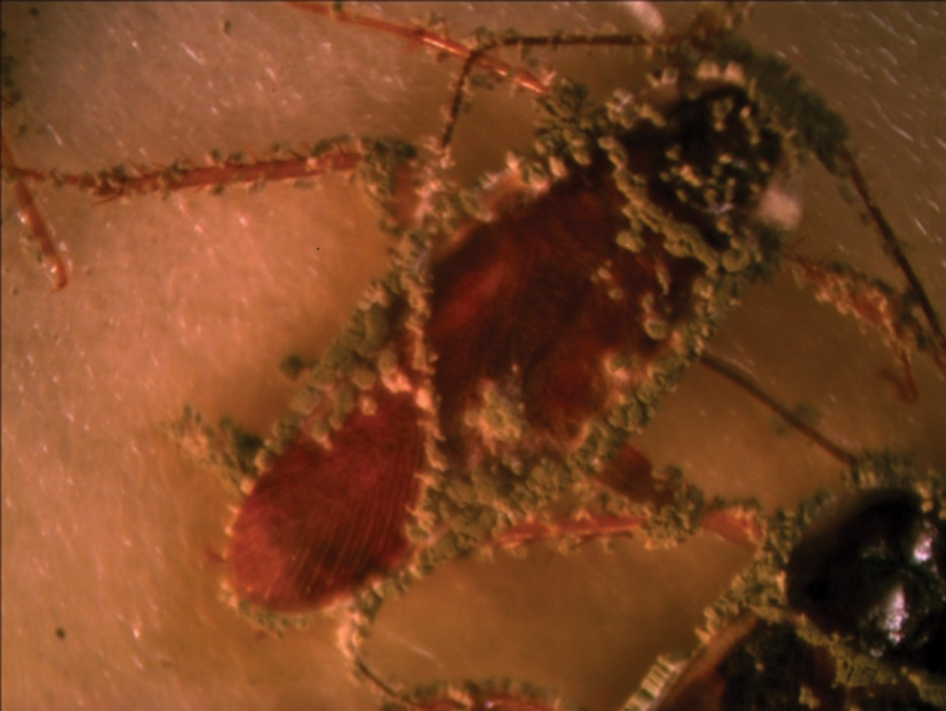
Male Cadaver With Green Muscardin

**Figure 2.**
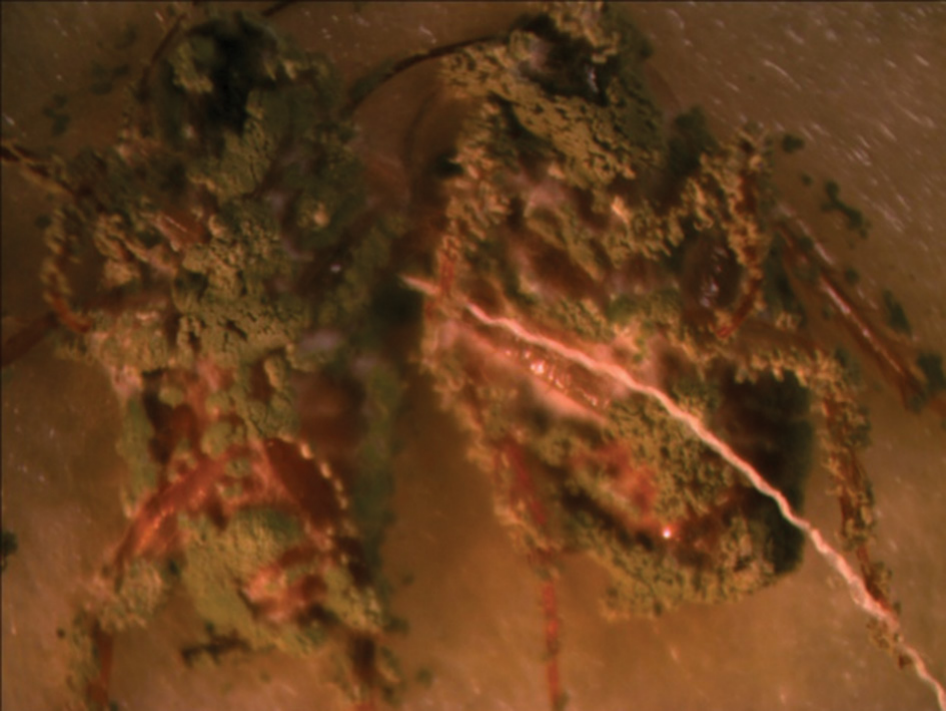
Male and Female With Green Muscrdin

Results of means comparison for nymph mortality showed significant differences between mortality percentages in all groups although the difference between the three final treatments was negligible, especially at seven days post exposure. LD_50_ and LD_90_ values were 4.5 × 10^6^ and 2.9 × 10^7^ on the third day but these values changed to 3.1 × 10^6^ and 3.1 × 10^6^ on the seventh day for nymphs. Survival times of nymphs were lower for those exposed to treated surfaces compared to untreated surfaces or the control group. The ST_50_ values reduced with an increased dose and the lowest survival time was observed for proportions of 25%, 50% and 100%, while the difference between their ST_50_ was not significant (Table 3). Comparison of the LD_50_ and LD_90_ confidence intervals (CI) for adults and nymphs showed an overlap of CI on the third day and seventh day, thus the differences between lethal dose values of adults and nymphs were not significant on the third day and seventh day (Table 2). All sterilized cadavers showed green muscardin on body surfaces. Mycelia growth and sporulation occurred in the intersegment region (Figures 1 and 2).

### 4.2. Bioassay 2: Treated Surface: Larger Scale (Semi-field)

Proportions of 10% and 25% were chosen as optimal doses for large-scale evaluation under room conditions (semi-field conditions) because results of laboratory tests indicated nearly no differences in mortality rates and survival times of cockroaches in these treatments. Mortality means of adults, adults and nymphs and nymphs were noticeable in both treatments and differed from those in the control groups. Mortality ranged from 77%-87% and from 89%-97% for proportions of 10% and 25%, seven days post exposure. Simultaneous mortality of cockroaches was noted in addition to higher mortality rate at proportion of 25% compared to 10% (Table 4). 

**Table 4.** Cumulative Mortality (%) of *S. longipalpa* Nymphs and Adults Exposed to Different Doses of *M. anisoplae* (IRAN 437C) Presented as Powder Formulation (At Room Conditions and on a Large Scale) ^a^

	Adults		Adults and Nymphs		Nymphs	
	Days After Inoculation					
	4 Days	7 Days	4 Days	7 Days	4 Days	7 DAYS
**Control**	3 ± 0.3	8 ± 0.9	2 ± 0.6	7 ± 0.7	1.3 ± 0.3	5 ± 0.3
**10% (6.6 × 10**^**6**^)	75 ± 1.5	87 ± 2	71 ± 1.2	83 ± 2.7	68 ± 1.8	77 ± 1.4
**25% (1.65 × 10**^**7**^)	87 ± 1.5	97 ± 0.68	81 ± 1.5	93 ± 2.1	73 ± 2.1	89 ± 1.2

^a^ Mean ± SD (Tukey’s test α = 0.05).

## 5. Discussion

 In the current study, dust formulation of *M. anisopliae* strain IRAN 437C was effective and caused high mortality in *S. longipalpa* nymphs and adults at seven days post exposure under laboratory conditions. Mortality rate of 25% Concentration of this isolate also caused over 80% in adult, adult and nymph and nymph populations in this time period under room conditions (semi-field condition). Although cockroaches were continuously exposed to treated surfaces in semi-field conditions, mortality percentages were lower than laboratory results. The main reason for this difference could probably be temperature fluctuations. Production of high-volume conidia, ability to grow in relatively low humidity and greater virulence of this isolate were contributing factors that explain why this isolate was selected to control the brown-banded cockroach. This isolate showed high efficiency in control of adults and larva of house fly among 10 Iranian isolates of *M. anisopliae* and *B. bassiaa* (18).

Conidia dust-formulation of *M. anisopliae* isolate ESALQ1037 at dose of 6.5 × 10^6^ conidia/cm^2^ with mineral powder talc as the carrier caused 73.9% and 96.9% mortality in nymphs and adults of *Blattella germanica*, nine days after exposure. The mortality rates varied from 76.1% to 100% after 15 days. Mean survival time (ST_50_) of nymphs and adult German cockroaches were 6.5 and 5.6 days, respectively at this dose (17). Using 3 × 10^7^ conidia/mL of *B. bassiana* and *T. harzianum* as inoculated bait against adults of *S. longipalpa* in Iraq resulted 86.67% and 36.67% mortality at seven days post exposure, respectively (18). In our study, mortality percentages of nymphs and adults of *S. longipalpa* were 82.5% and 85% at seven days post treatment at similar doses (proportion of 10% ≈ 6.6 × 10 conidia/cm^2^) and survival times were 4.1 and 3.9 days, respectively. The observed differences in mortality rates and survival times could be attributed to cockroach species, fungal isolates, duration of the study and the experimental conditions. *S. longipalpa* adults were more susceptible than nymphs to *M. anisopliae* infection because there was always higher mortality observed for adult treatments than nymphs at the same dose. This difference in susceptibility could be related to cuticle molting by nymphs, particularly when ecdysis occurs immediately after a pathogen inoculation or when the time interval between ecdysis is short (17, 19). Spiracle blocking by fungal conidia could be another reason contributing to higher mortality rates in adults. It seems that molting and ecdysis in the tracheal system of nymphs decreases the probability of spiracle blocking and reduces the rate of nymph mortality (20).

**Figure 3.**
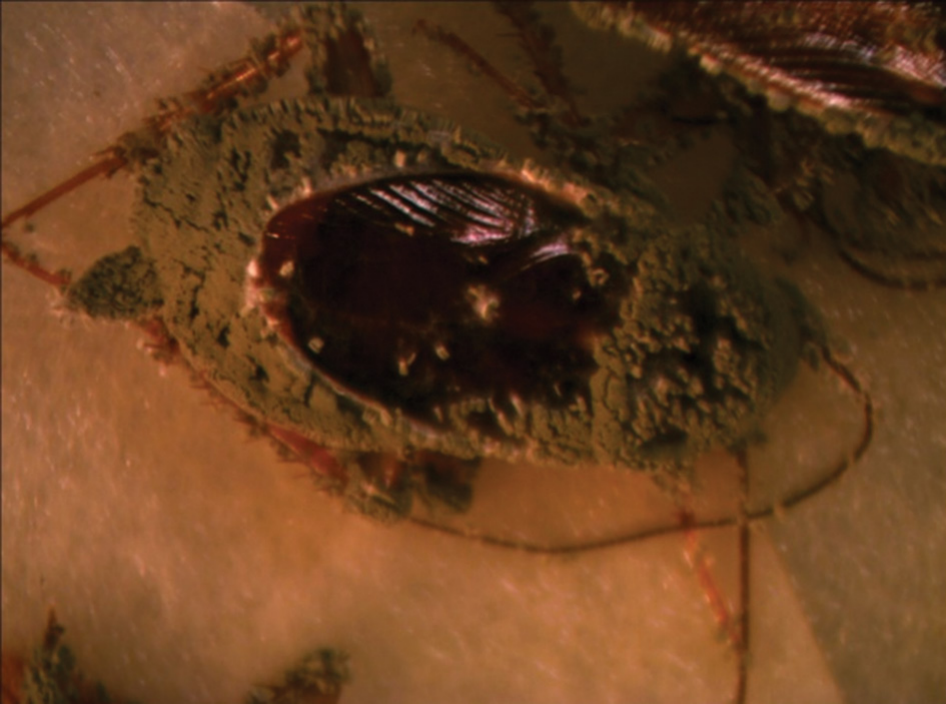
Female Cadaver With Green Muscardin

**Figure 4.**
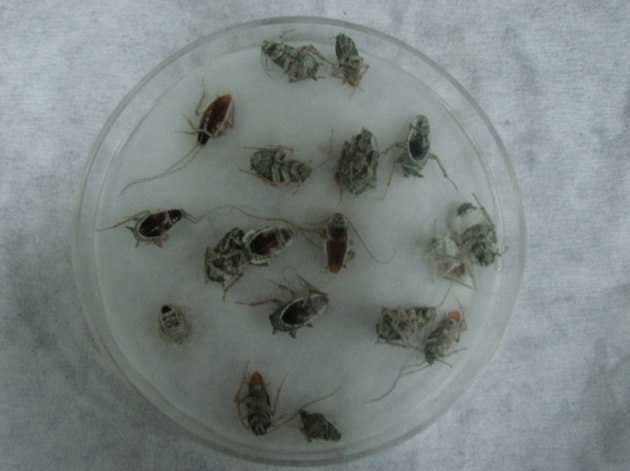
Cockroach Cadavers on Damp Filter Paper With Muscardin Symptoms

Although the cuticle of insects constitute an important physical barrier for protection against penetration of entomopathogenic fungal conidia, high mortality rates were observed when adults were exposed to powder formulations. Intersegment regions of the thorax and abdomen, mouthparts and legs (Figures 1 -4) as favorable areas for conidia adherence are more difficult to clean and lead to conidia penetration and adult contamination (17).

Use of microbial control agents is an appropriate alternative to chemical insecticides as they are safe to humans and lead to a reduction in the large volume of chemicals used universally to control cockroaches. They also have an ability to cause high mortality rates in cockroach populations. The speed of cockroach killing is an important factor when considering entomopathogenic fungi as a bio-control agent. Mortality of adults and nymphs of the brown-banded cockroach usually began on the third day and reached 100% on the seventh day after exposure to dust-formulation of *M. anisopliae*. Although there is a time lag between the application of treatment and the onset of cockroach mortality compared with the more rapid effect of chemical insecticides, this time lag could be reduced by application of a combination of entomopathogenous fungi with sub lethal doses of an insecticides (19). This time lag may also be acceptable where cockroaches have become resistant to chemicals that are no longer effective against them.

In conclusion, results of this study indicated that application of dust-formulation of *M. anisopliae* Iran 437C has good potential to control adults and nymphs of the brown-banded cockroach. This is promising as an effective alternative for myco-insecticide against cockroaches and the house fly (11, 13), but more researches are necessary to determine the efficacy of this fungal isolate on a larger scale and under natural conditions.
